# A large-scale in vivo screen to investigate the roles of human genes in *Drosophila melanogaster*

**DOI:** 10.1093/g3journal/jkae188

**Published:** 2024-08-09

**Authors:** Ashley Avila, Lily Paculis, Roxana Gonzalez Tascon, Belen Ramos, Dongyu Jia

**Affiliations:** Department of Biology, Georgia Southern University, Statesboro, GA 30460, USA; Department of Biology, Georgia Southern University, Statesboro, GA 30460, USA; Department of Biology, Georgia Southern University, Statesboro, GA 30460, USA; Department of Biology, Georgia Southern University, Statesboro, GA 30460, USA; Department of Biology, Georgia Southern University, Statesboro, GA 30460, USA; Department of Molecular and Cellular Biology, Kennesaw State University, Kennesaw, GA 30144, USA

**Keywords:** screen, human genes, *Drosophila*, eye

## Abstract

Understanding the signaling pathways in which genes participate is essential for discovering the etiology of diseases in humans. The model organism, *Drosophila melanogaster*, has been crucial in understanding the signaling pathways in humans, given the evolutionary conservation of a significant number of genes between the two species. Genetic screens using *Drosophila* are a useful way of testing large number of genes to study their function and roles within signaling pathways. We conducted a large-scale genetic screen to identify which human genes cause an alteration in the morphology of the *Drosophila* eye. The *GMR-Gal4* was employed to activate a single *UAS*-human gene in the eye tissue. In total, we screened 802 *UAS*-human gene stocks, corresponding to 787 human protein-coding genes, for the ability to influence eye development. We found that overexpression of 64 human genes were capable of disrupting eye development, as determined by phenotypic changes in eye texture, size, shape, bristle morphology, and ommatidia organization. Subsequent analysis revealed that the fly genome encodes proteins that are homologous to a majority of the 64 human genes, raising the possibility that overexpression of these transgenes altered eye development by altering the activity of evolutionarily conserved developmental signaling pathways. Consistent with this hypothesis, a secondary screen demonstrated that overexpression of fly homologs produced phenotypes that mimicked those produced by overexpression of the human gene. Our screening has identified 64 human genes capable of inducing phenotypes in the fly, offering a foundation for ongoing research aimed at understanding functionally conserved pathways across species.

## Introduction

The fruit fly, *Drosophila melanogaster*, has proven to be a powerful and reliable model to study evolutionarily conserved genes with known roles in human disease. As nearly 75% of human disease-associated genes have a homolog in *Drosophila*, studies in the fly can rapidly identify genes that contribute to disease-related phenotypes and efficiently study the molecular mechanisms by which conserved genes contribute to human health and disease progression (Reiter *et al*. 2001; Wangler *et al*. 2017). For instance, the Toll and Hedgehog pathways were originally discovered in *Drosophila* and later found to be evolutionarily conserved in humans, where they are involved in a variety of disease etiologies (Hahn *et al*. 1996; Rock *et al*. 1998; Mullor *et al*. 2002; Cook *et al*. 2004). Considering that the fly has a short life cycle, is relatively inexpensive to culture, and the fly community has generated a massive collection of genetic tools (Roote and Prokop 2013; Tolwinski 2017), the genetic similarities between *Drosophila* and humans position the fly in an ideal position to conduct large-scale genetic screens.

The *Drosophila* eye has a highly organized structure that allows alterations to be easily observed, making it an ideal system for genetic screening. The eye is composed of around 750 ommatidia that are organized symmetrically and are separated by eye bristles (Ready *et al*. 1976). About two-thirds of the genes in the *Drosophila* genome contribute to the development of the fly's eye, which indicates that many of these same genes also have functions in tissues beyond the eye (Iyer *et al*. 2016). Moreover, the fly community has generated several genetic tools for manipulating gene expression in the eye, with the driver *GMR-Gal4* a powerful genetic tool capable of specifically expressing UAS transgenes within eye cells. Specifically, the *GMR* promoter activity is localized to the cells positioned behind the morphogenetic furrow in the third instar larval eye disc, where they have ceased dividing and initiated the process of differentiation (Freeman 1996).

Previous studies have used the *GMR-Gal4/UAS* system to express human genes in the *Drosophila* eye (Xu *et al*. 2008; Katanaev *et al*. 2020). These studies demonstrated that expressing human genes in the eye is able to produce phenotypic changes. The ability of these exogenous genes to induce abnormal tissue formation in the fly eye points to possible functionally homologous gene products or atypical functions such as dominant negative effects (Prelich 2012). Phenotypic changes in eye texture previously documented encompass the rough eye and glossy eye phenotypes. The rough eye phenotype is characterized by a reduced eye size and disorganized ommatidia (Iyer *et al*. 2016). In the glossy phenotype, a reflective appearance of the eye emerges due to the loss of antireflective nipple arrays (Kryuchkov *et al*. 2011).

Here, we exploit an existing *Drosophila* genetic collection to determine how 802 UAS transgenes that drive expression of 787 human genes interfere with eye development. We found that expression of 64 of these transgenes induced visible phenotypes, including morphological changes in eye texture, size, shape, as well as ommatidia organization, and bristle morphology. Moreover, subsequent validation studies revealed that overexpression of the fly homologs of these genes often induced similar phenotypes, highlighting the conserved molecular function of these essential genes. Overall, our study provides a resource for any lab in the *Drosophila* genetics community interested in studying human gene function within the context of fly development.

## Materials and methods

### Fly strains and husbandry

*GMR-Gal4* (BDSC #1104) is a gift from Dr. Wu-Min Deng laboratory, *UAS*-human (Supplementary Table 1) and *UAS*-fly (Supplementary Table 5) gene stocks used for this experiment were obtained from Bloomington *Drosophila* Stock Center. The UAS-human gene stocks for the screen were acquired from a collection at the Bloomington Stock Center (https://bdsc.indiana.edu/stocks/uas/uas_hsap.html), which compiles UAS lines that express human proteins or protein variants. These stocks are from different collections/laboratories. We unbiasedly tested these stocks based on the alphabetical order of gene names, if multiple lines existing for one gene, we randomly selected one. Flies were maintained with standard Bloomington fly food (39 L H_2_O, 225 g agar, 675 g dried deactivated yeast, 2850 g cornmeal, 3 L light corn syrup, and 188 mL propionic acid) in 25°C incubators.

### Genetic screen and eye phenotype assessment

During the screen, only Bloomington numbers were assigned to UAS-human flies to avoid performance bias. The *GMR*-*GAL4* line was crossed with UAS-human gene lines, resulting in F1 progeny carrying the *GMR-GAL4*/*UAS* binary expression system. For each gene, 10 flies composed of equal males and females were screened for severe phenotypes using a stereo microscope. Then, three male and female flies were imaged using SEM. Using images acquired from stereo microscope and SEM, the eyes were visually assessed based on the size (normal, smaller, and larger), texture (rough, glossy, and rough/glossy), and shapes of the eyes (normal, circular, lobe, kidney, elliptical, and raindrop shapes). The fly homologs selected for screening were chosen based on the NCBI Protein Blast expect value (*e*-value). The *e*-value signifies the probability that a given sequence alignment is due to random chance rather than indicating a biological connection between sequences. It diminishes exponentially with higher match scores. A lower *e*-value signifies a more statistically significant match.

### Image acquirement

Flies underwent screening for alterations in eye phenotype using CO_2_ anesthesia, followed by examination with a Leica M60 Stereo microscope. Positive hits were imaged using a Leica EC4 Digital Camera at 50× magnification and a JEOL JSM-6610LV scanning electron microscope (SEM) at 100× and 1,000× magnification. Fly genes were screened using Olympus SC180 digital camera to capture light images and Tescan Vega 3 scanning electron microscope for SEM images due to lab relocation. Preparation of flies for SEM imaging was followed as previously described (Ohta *et al*. 2014). The images were cropped by Image J to display the regions of interest, and assembled in Photoshop.

### Bioinformatics

Databases that were used to perform data analysis of the genes, which produced a phenotypic change, include prediction of clinical outcomes from genomic profiles (PRECOG), DRSC integrative ortholog prediction tool (DIPOT), and NCBI protein blast. Information from databases was collected April 2023. PRECOG is a database that contains gene expression data from patients with 39 different malignancies (Gentles *et al*. 2015). It provides information as to whether a gene's high expression in a type of cancer is associated with poor or good survival outcomes. Genes that produced a cancer survival outcome *Z*-score of ≥3.00 or ≤−3.00 were grouped based on the type of cancer associated. A positive *Z*-score represented poor survival and a negative *Z*-score with a good survival outcome. DIOPT Version 8 was used to find the most homologous fly gene for the 64 human genes that produced a change in phenotype (Hu *et al*. 2011). If a human gene had several fly homologs, the gene with the highest weighted score was selected. In cases where a human gene had several fly homologs with the same weighted score, a single fly homolog gene was randomly selected. NCBI Protein Blast was then used to compare amino acid structure between the protein product from the 64 *Homo sapiens* genes and the *Drosophila melanogaster* homolog equivalents found using DIOPT (Altschul *et al*. 1990). We utilized GeneCards Version 5.19 to gather comprehensive information about the 64 human genes, while for gene information concerning the fly homologs, we referred to FlyBase version FB2024_01 (Gramates *et al*. 2022; Öztürk-Çolak *et al*. 2024).

## Results

### A large-scale screen of human genes in *Drosophila*

**Fig. 1.**
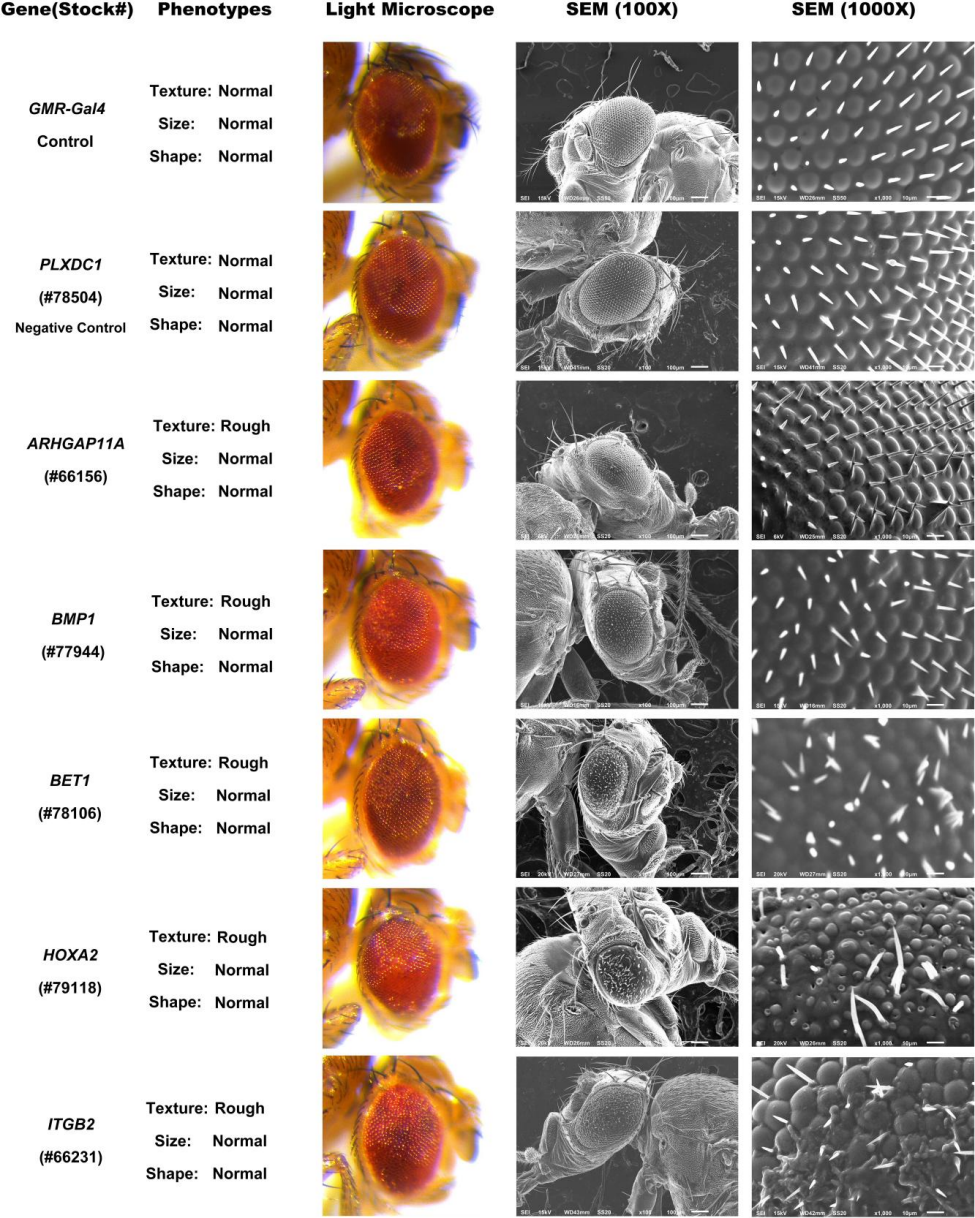
Developmental defects caused by overexpression of human genes in the *Drosophila* eye. Images depicting the phenotypes caused by *GMR*-*Gal4* induced expression of UAS transgenes in the developing fly eye. When compared with the *GMR-Gal4* negative control, expression of the listed human genes results in a rough eye phenotype. For all transgenes, eyes were imaged using both light microscopy and SEM. The scale bar for SEM (100×) images is 100 µm. The scale bar for SEM (1000×) images is 10 µm. The BDSC stock number for each strain is listed in parentheses underneath the gene name.

**Fig. 2.**
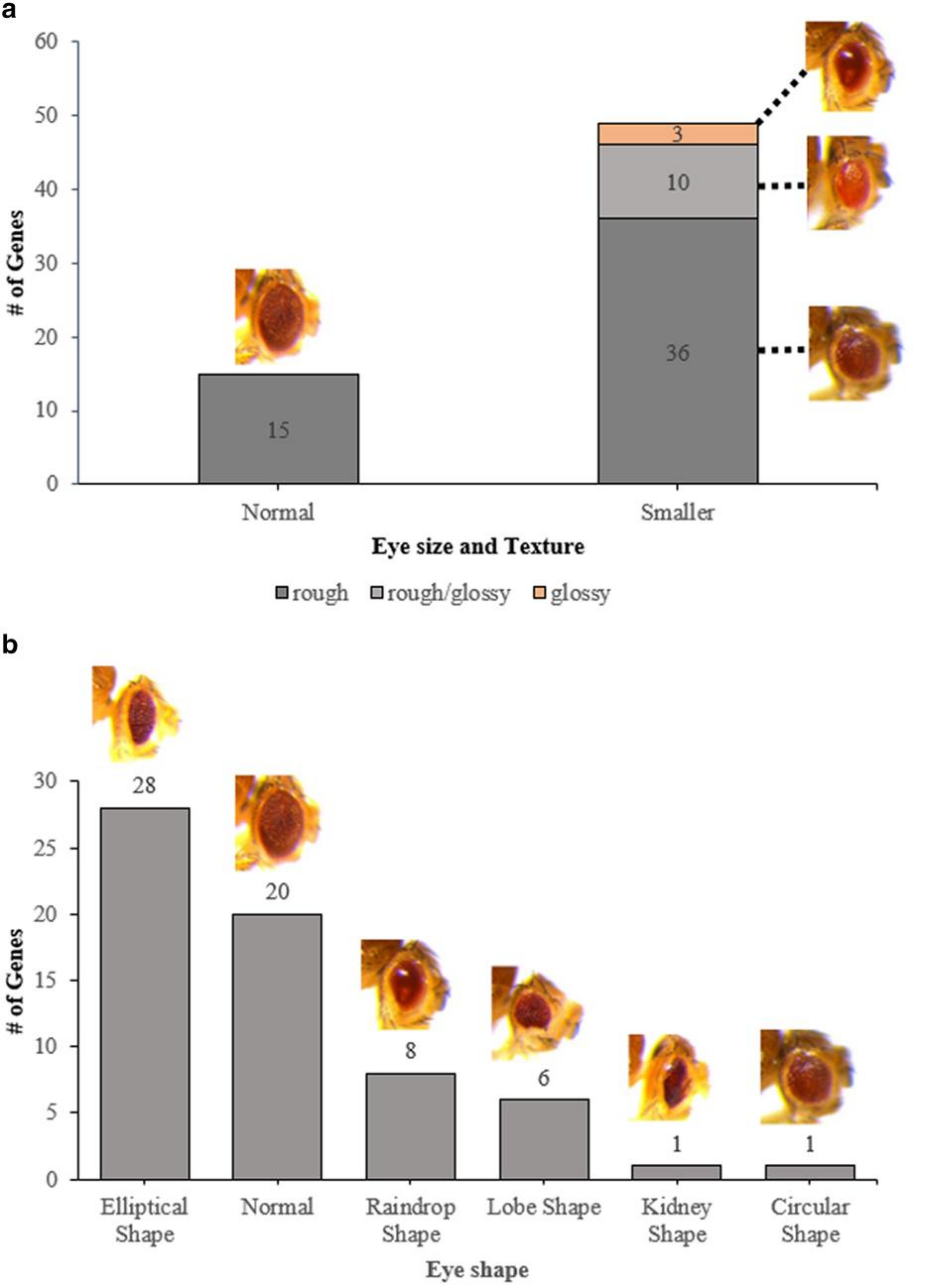
Phenotypic changes induced by expression of 64 human genes in the *Drosophila* eye. A graphical representation of the phenotypes observed among of the 64 human genes identified as positive hits in the overexpression screen. *GMR-Gal4* induced expression of most of these transgenes induced a) small eye phenotypes, rough eye phenotypes, and b) eyes with an elliptical shape.

**Fig. 3.**
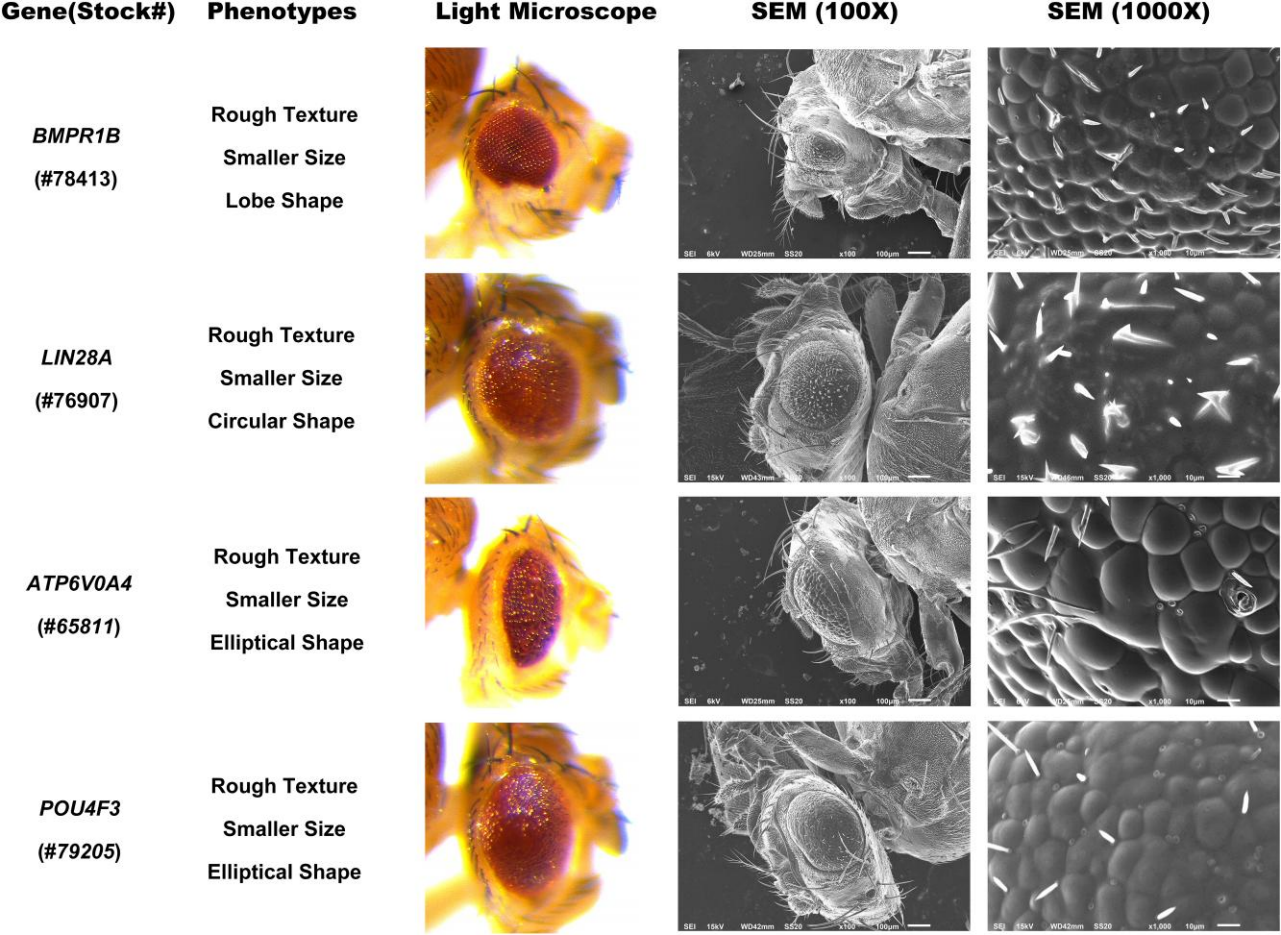
Representative images of the small eye phenotype induced by human transgene expression. For all transgenes, eyes were imaged using both light microscopy and SEM. The scale bar for SEM (100×) images is 100 µm. The scale bar for SEM (1000×) images is 10 µm. The BDSC stock number for each strain is listed in parentheses underneath the gene name.

**Fig. 4.**
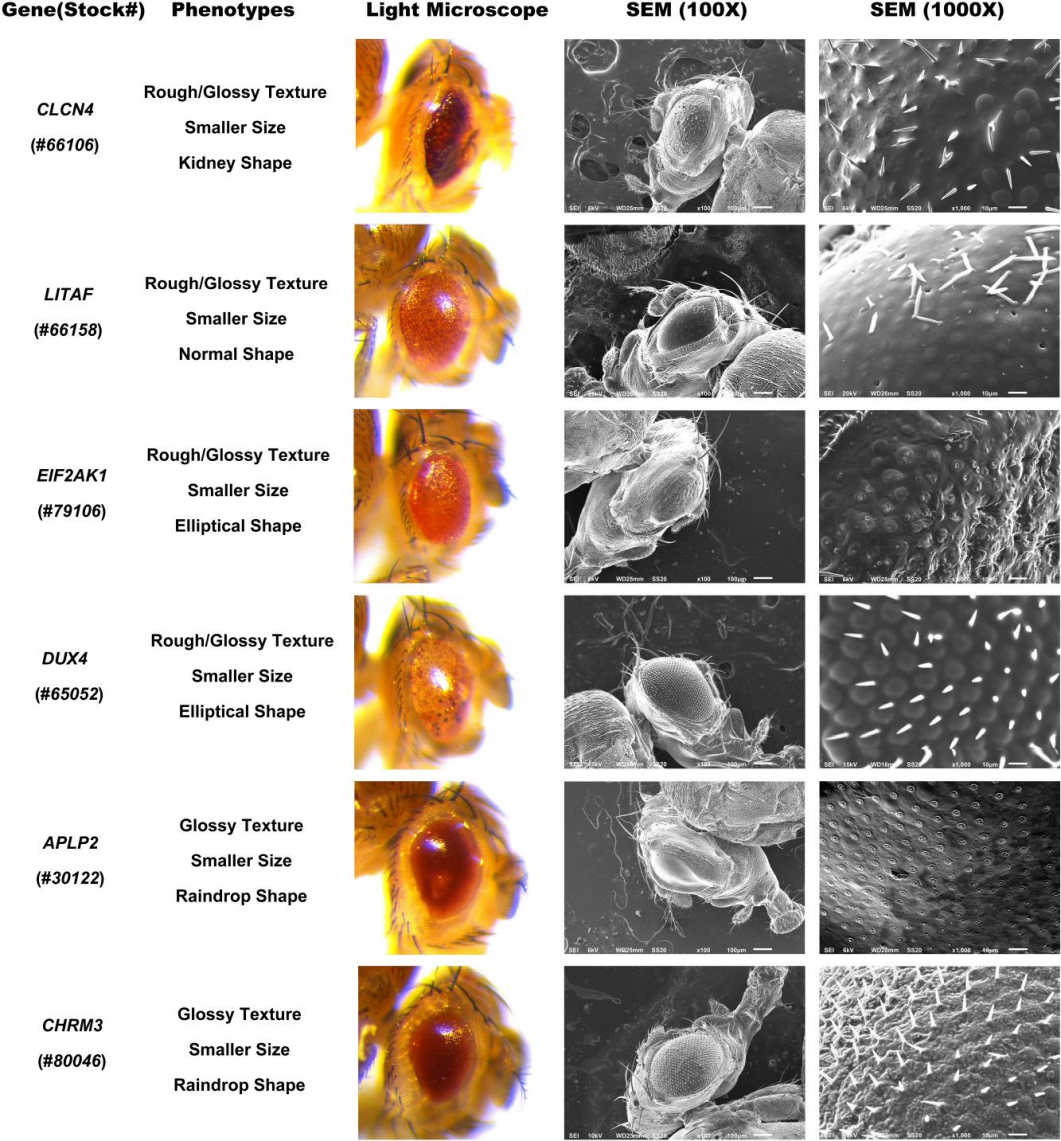
Representative images of the eye texture and shape phenotypes induced by human transgene expression. For all transgenes, eyes were imaged using both light microscopy and SEM. The scale bar for SEM (100×) images is 100 µm. The scale bar for SEM (1000×) images is 10 µm. The BDSC stock number for each strain is listed in parentheses underneath the gene name.

In total, 802 *UAS*-human gene stocks were screened by crossing the *GMR*-*Gal4* driver with the *UAS*-human gene lines. This resulted in the screening of 787 human genes, factoring in the redundancy within the initial pool of 802 human stocks (Supplementary Table 1). Of those 802 stocks tested, 64 stocks produced altered eye phenotypes making up 8.0% of the total (Supplementary Tables 1 and 2). The rest of the human genes tested produced no visibly obvious phenotypic changes in the fly eye that could be easily detectable under a light microscope. There were no notable differences between males and females of the same genes. For the reference of normal phenotypes, *GMR-Gal4* and *GMR-Gal4*> plexin domain containing 1 (*PLXDC1*) were used, as they did not induce noticeable phenotypic changes (Fig. 1). All 64 identified human gene stocks showed a change in eye texture, with 51/64 displaying a rough eye phenotype (Fig. 2a). As seen in Figs. 1 and 3, the misexpression of these genes caused partial or full rough texture of the eyes. Among the 64 genes, 10 were classified as rough/glossy phenotypes because they induced both a rough texture and a glossy appearance in the eye, whereas 3 genes led to the glossy eye phenotypes (Figs. 2a and 4).

Further examination was conducted on the size of the eyes, revealing either no alteration or a decrease in size. In total, 15/64 had normal size eyes, and 49/64 showed smaller sizes (Fig. 2a). Another notable change in phenotypes was those that affected the overall shape of the eyes. The variety of shapes observed included normal, circular, lobe, kidney, elliptical, and raindrop shape (Fig. 2b). For instance, *GMR-Gal4* induced expression of bone morphogenetic protein receptor type 1B (*BMPR1B*) resulted in a lobe eye shape, characterized by a ventral notch in the eye (Fig. 3). Expression of the gene lin-28 homolog A (*LIN28A*) let to a circular eye shape that was distinguished by its roundness compared to the oval shape (Fig. 3). Similarly, *GMR-Gal4* induced expression of ATPase H+ transporting V0 subunit a4 (*ATP6V0A4*), POU class 4 homeobox 3 (*POU4F3*), and double homeobox 4 (*DUX4*) induced changes in eye shape, with expression of all three genes resulting in eyes that were significantly elongated and stretched when compared controls (Figs. 3 and 4). The gene, chloride voltage-gated channel 4 (*CLCN4*) resulted in a kidney eye shape with a notch on the side (Fig. 4). Finally, we also observed that expression of amyloid beta precursor like protein 2 (*APLP2*) and a subset of other transgenes caused the eye to develop into raindrop shape (Fig. 4).

Under low magnification overexpression of some genes fails to induce obvious changes in eye shape and size. However, SEM microscopy revealed that the various human transgenes induced distinct bristle and ommatidia phenotypes (Fig. 1). When compared with the control eye, expression of the following human genes induced distinct phenotypes: (1) Rho GTPase activating protein 11A (*ARHGAP11A*) resulted in misaligned bristles. (2) Bone morphogenetic protein 1 induced an array of phenotypes, including bristle loss, bristle shortening, disrupted hexagonal tile patterning, and irregularly shaped ommatidia. (3) Eyes expressing Bet1 Golgi vesicular membrane trafficking protein (*BET1*) exhibited loss of a few bristles, two or multiple bristles at some locations, and disrupted hexagonal tile pattern with many irregular shaped ommatidia. (4) Homeobox A2 resulted in loss of most bristles and disrupted hexagonal tile pattern with many irregular shaped ommatidia. (5) Integrin subunit beta 2 showed loss of some bristles, and a portion of ommatidia displaying a wrinkled surface.

We also observed a number of distinct defects among those genes whose expression induced a rough and smaller eye phenotype (Fig. 3): (1) *BMPR1B* displayed a reduction in bristles, accompanied by a disruption of the hexagonal tile pattern and some irregularly shaped ommatidia. (2) *LIN28A* showed loss of some bristles, two or multiple bristles at some locations, some bristles shorter, disrupted hexagonal tile pattern, and some irregular shaped ommatidia. (3) *ATP6V0A4* displayed loss of many bristle shafts with socket remaining, and disrupted hexagonal tile pattern with large ommatidia. (4) *POU4F3* exhibited loss of many bristle shafts with sockets left, multiple bristle sockets at some locations, and disrupted hexagonal tile pattern with some irregular shapes.

A number of genes caused either rough/glossy or glossy small eye phenotypes (Fig. 4). The ommatidia in these phenotypes display varying degrees of fused ommatidia, with the exception of *DUX4*. Among those genes: (1) *CLCN4* showed loss of many bristles, a disrupted bristle pattern, and irregularly shape ommatidia. (2) The gene, lipopolysaccharide induced TNF factor, resulted in loss of many bristles, and almost invisible ommatidia with a smooth surface. (3) Eukaryotic translation initiation factor 2 alpha kinase 1 displayed loss of bristle shafts with socket remaining, no visible ommatidia, and a wrinkled surface. (4) *DUX4* exhibited the loss of a few bristles and a glossy appearance due to the loss of eye pigment, without affecting the ommatidia. (5) *APLP2* presented with bristle shafts missing with socket remaining and fused ommatidia, creating a smooth surface. (6) Cholinergic receptor muscarinic 3 exhibited less defined ommatidia with a wrinkled surface.

In general, human genes that disrupted the formation of eye development had a tendency to reduce the size of the eye, cause a rough eye phenotype, and result in an elliptical-shaped eye. When only the rough phenotype is present, the resulting phenotypes are weaker. However, when both the rough and smaller eye phenotypes are present, the phenotypic changes tend to be more profound.

### Prediction of clinical outcomes from genomic profiles

**Fig. 5.**
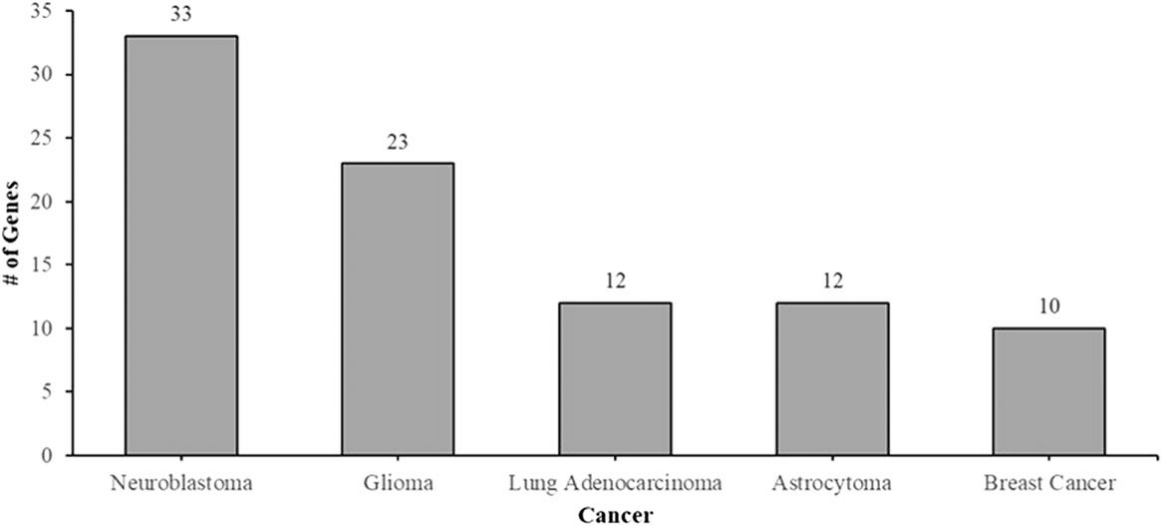
The human genes that induce abnormal eye phenotypes are associated with cancers of the nervous system. Cancer survival outcome data from PRECOG reveals that 32 of the human genes isolated from the screen are associated with neuroblastoma. Three of the five cancers are associated with the nervous system.

Next, we investigate whether our target genes from the screen are potentially linked with human diseases, the PRECOG database with gene expression data from 39 malignancies was utilized to examine the gene hits and their associated cancer survival outcomes. The 64 genes in our set were associated with 22 out of the 39 malignancies on PRECOG. Additionally, 40/64 genes were linked to two or more cancers, with glycogen phosphorylase B (*PYGB*) and lamin B1 (LMNB1) having the most associations with seven cancer types (Supplementary Table 3). The top three cancers that the set of genes were found to be associated with were neuroblastoma, glioma, and lung adenocarcinoma (Fig. 5).

### DIPOT and NCBI protein blast

To explore protein homology between the human genes in the hit set and the predicted homologs in Drosophila, we used both DIPOT and NCBI Protein BLAST. The majority of the human genes examined had predicted *Drosophila* homologs on DIPOT. However, no fly homologs were identified for the human genes caveolin 3, high mobility group AT-hook 1 (*HMGA1*), and protein tyrosine phosphatase receptor type C associated protein (*PTPRCAP*) (Supplementary Table 4). A comparison of human and fly gene protein products revealed varying degrees of protein similarity through protein alignment using NCBI protein blast. As shown in Supplementary Table 4, the human gene, protein phosphatase 1 catalytic subunit alpha (*PPP1CA*), exhibited high levels of protein similarity with the fly homolog *Protein phosphatase 1* at 87B (Pp1-87B). *PPP1CA* and *Pp1-87B* displayed a significant alignment match of 90% as indicated by the query coverage. Within the aligned portions of the protein sequences, 90.26% of the residues were identical.

### *Drosophila* homolog overexpression

In our analysis, we employed the *e*-value to assess the similarity between the human gene and its nearest fly homolog. Genes exhibiting both highly conserved and less conserved structures were chosen. We examined 11 homolog fly genes, each displaying varying degrees of conservation with their human counterparts (Supplementary Table 5). The three most conserved genes in our list, *Glycogen phosphorylase* (*GlyP*), *patched* (*ptc*), and *sticks and stones* (*sns*), have lowest *e*-values 0, 3.00E-144, 1.00E-130 with their human gene counterparts *PYGB*, patched 1 (*PTCH1*), and NPHS1 adhesion molecule, nephrin (*NPHS1*), respectively. The least conserved gene in our list is *Dab*, which has a high *e*-value 6.70E-01 with its human gene counterpart complement C8 alpha chain (*C8A*).

**Fig. 6.**
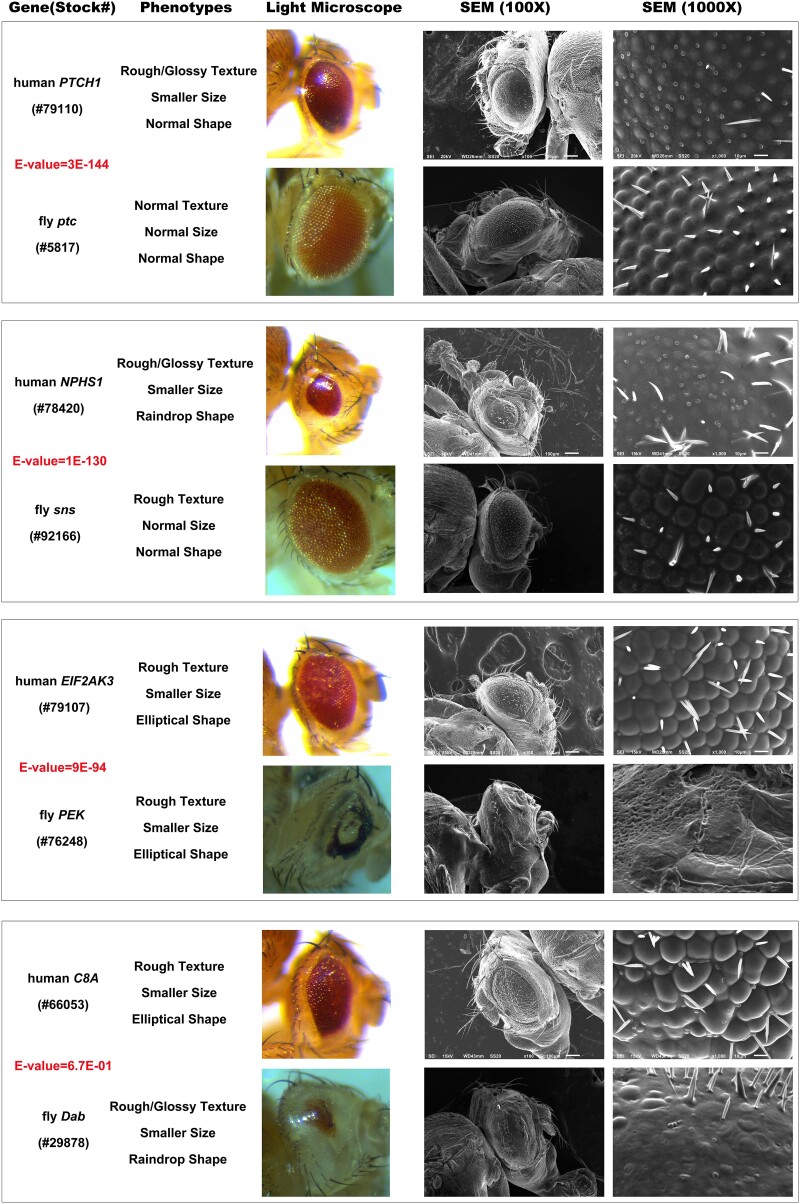
Images depicting eye phenotype comparisons between human and fly gene overexpression. For all transgenes, eyes were imaged using both light microscopy and SEM. The scale bar for SEM (100×) images is 100 µm. The scale bar for SEM (1000×) images is 10 µm. The BDSC stock number for each strain is listed in parentheses underneath the gene name. NCBI Protein Blast expect value (*e*-value) is provided.

Our experimental results demonstrated that overexpression of all the 11 fly homolog genes showed similar phenotypes with their human counterparts, including those less conserved (Supplementary Table 5, Fig. 6). For instance, overexpression of human gene *PTCH1* caused smaller eyes, loss of most eye bristles, disrupted hexagonal tile pattern with some irregular shapes. In comparison, the overexpression of *ptc* caused a weaker phenotype, including loss of a few bristles, slightly disrupted hexagonal tile pattern, and a few ommatidia with two bristles (Supplementary Table 4, Fig. 6). Another highly conserved human gene *NPHS1*'s overexpression led to rough/glossy eye texture, smaller eye size, raindrop eye shape. SEM further revealed loss of many eye bristle shafts with socket left, multiple bristle sockets at some locations, and almost invisible ommatidia with smooth surfaces. Overexpression of the fly homolog, *sns*, resulted in a weaker phenotype, presenting rough eye texture, loss of many eye bristles, two bristles at some locations, and ommatidia starting to have irregular shapes/smooth surfaces (Supplementary Table 5, Fig. 6). In general, overexpression of fly homolog genes generally produced weaker phenotypes, with a few notable exceptions. For instance, the overexpression of human eukaryotic translation initiation factor 2 alpha kinase 3 (*EIF2AK3*) resulted in rough eye texture, smaller eye size, elliptical eye shape, loss of some eye bristles, two or multiple bristles at some locations, disrupted hexagonal tile pattern with some irregular shapes, and a few fused ommatidia. The overexpression of its fly counterpart, *pancreatic eIF-2α kinase* (*PEK*), produced a much stronger phenotype, including rough eye texture, smaller eye size, elliptical eye shape, strongly apoptotic eye tissues with totally disrupted structure, and loss of all bristles (Supplementary Table 5, Fig. 6). In addition, less conserved fly genes not only showed similar phenotypes with their human counterparts but also can produce stronger phenotypes. For instance, the least conserved fly gene *Disabled* (*Dab*) overexpression had rough/glossy eye texture, smaller eye size, raindrop shape, loss of some bristle shafts with socket left, multiple bristle sockets at some locations, and disrupted hexagonal tile pattern with many irregular shapes. Its human counterpart *C8A* showed similar phenotypes, but to a much less extent (Supplementary Table 5, Fig. 6). These findings might suggest that the genes functioning across species are likely highly conserved during evolution, and our screen could provide candidate genes to study their functions across species.

### Eye development and function

Alteration in the *Drosophila* eye as a result of overexpressing either human gene or fly homolog suggests a conserved function. When gene overexpression functions were compared between the 11 screened fly genes and their corresponding human homologs, most had conserved gene functions (Table 1). The exceptions were the gene pairs 5-hydroxytryptamine receptor 1D (*HTR1D*) and octopamine-tyramine receptor (*Oct-TyrR*), as well as *C8A* and *Dab*, which exhibited different gene functions between the two species (Table 1). For the genes that had similar functions between species, it could suggest that the human gene led to alteration in eye development as a result of conserved protein function. In Table 1, we can see that previous research on the human genes *PTCH1*, *EIF2AK3*, and *APLP2*, along with their fly homologs show they are involved in eye development and function. The mutants of the fly homolog of *PTCH1*, known as *ptc*, have been observed to have changes in larval eye imaginal discs (Ma and Moses 1995) Moreover, in adults, different types of *ptc* trans-heterozygotes mutants show various degrees of alterations in eye size, shape, and texture (Thomas and Ingham 2003). *PEK* the fly homolog of *EIF2AK3* has been shown to reduce eye size and induce loss of ommatidia organization when overexpressed using *GMR*-*Gal4* (Malzer *et al*. 2010). In an *amyloid protein precursor-like* (*Appl*) fly mutant, it has been observed that they exhibit less preference for UV light compared to wild-type flies, which typically prefer UV light over visible light. Despite this change in preference no severe change in phenotype is seen in the eye (Mora *et al*. 2013).

**Table 1.** Eye development and function between *Drosophila* homologs and *Homo Sapiens*.

Homology (*e*-value)	Gene	Organism	Function	Eye development/function refs
0	*PYGB*	Human	Glycogen phosphorylase	N/A
	*GlyP*	Fly	Glycogen phosphorylase	N/A
3E−144	*PTCH1*	Human	Receptor for hedgehog ligands	(Chassaing *et al*. 2016; Cavodeassi *et al*. 2019)
	*ptc*	Fly	Hedgehog receptor	(Ma and Moses 1995; Strutt and Mlodzik 1996; Thomas and Ingham 2003)
1E−130	*NPHS1*	Human	Cell adhesion molecule -immunoglobulin family	N/A
	*sns*	Fly	Surface receptor-immunoglobulin superfamily	(Bao 2014)
9E−94	*EIF2AK3*	Human	Kinase	(Saptarshi *et al*. 2022)
	*PEK*	Fly	Kinase	(Malzer *et al*. 2010)
3E−53	*DRD2*	Human	Dopamine receptor	(Caravaggio *et al*. 2018)
	Dop2R	Fly	G-protein coupled receptor/dopamine receptor	N/A
1E−46	*HTR1D*	Human	G-protein coupled receptor/serotonin receptor	N/A
	*Oct-TyrR*	Fly	G-protein coupled receptor/tyramine receptor	N/A
2E−37	*APOD*	Human	A component of high-density lipoprotein	N/A
	*Nlaz*	Fly	Lipid binding protein	N/A
2E−35	NXF5	Human	Export of mRNA from nucleus to cytoplasm	N/A
	*sbr*	Fly	Export mRNA from nucleus to cytoplasm	(Mamon *et al*. 2021)
6.00E−35	*APLP2*	Human	Transmembrane protein-amyloid precursor protein family	(Tkatchenko *et al*. 2015)
	*Appl*	Fly	Transmembrane protein-beta amyloid-like protein	(Mora *et al*. 2013)
1E−21	*ASCL1*	Human	BHLH transcription factor	(Wohlschlegel *et al*. 2023)
	ac	Fly	BHLH transcription factor	N/A
0.67	*C8A*	Human	Component of the complement system	N/A
	*Dab*	Fly	Adaptor protein	(Le and Simon 1998)

Some genes with conserved functions were found to be involved in the eye of one organism but not the other, such as the human genes Dopamine Receptor D2 (*DRD2*) and Achaete-Scute Family BHLH Transcription Factor 1 (*ASCL1*), and the fly genes *small bristles* (*sbr*) and *sns*. In *sbr* mutant larvae it was noted that there was defective photoreceptor axon bundling (Mamon *et al*. 2021). The other fly gene, *sns* in combination with another gene *hibris* (*hbs*) is needed for spatial organization of primary pigment cells in the eye (Bao 2014). Other conserved functional gene pairs such as Glycogen Phosphorylase B (*PYGB*) and *GlyP*, as well as apolipoprotein D (*APOD*) and *Neural Lazarillo* (*Nlaz*), have not been demonstrated to be involved in eye development or function. Genes with known functions in the eye, either in both species or in one species, may facilitate further research in discovering new pathways more easily compared to genes with no known involvement in the eye.

## Discussion

Here, we describe a genetic screen to identify human genes that are capable of interfering with normal *Drosophila* eye development. Overall, we found 64 human genes capable of disrupting normal development and cellular function in the *Drosophila* eye—a positive hit rate 8%, which is similar to the findings of a similar eye screen study (Xu *et al*. 2008), where overexpression of human genes resulted in a 10% positive hit rate. The positive hit rate in our screen, therefore, was within expected levels.

Beyond the usefulness of determining which human genes induce developmental phenotypes, our study also produced a number of interesting findings. Our observation that each UAS transgene induced a unique eye phenotype suggests that these 64 transgenes will prove a valuable reagent for future mechanistic studies. Moreover, we also made the exciting observations that both the extent of homology between the human gene and fly gene varied among these 64 hits. For instance, the gene *PPP1CA* was found to have a homolog with a high degree of similarity, while *HMGA1* lacked a known homolog in *Drosophila* (Supplementary Table 3). We also demonstrated the utility of this approach by demonstrating that phenotypes induced by overexpression of a human gene are predictive of the phenotype induced by overexpression of the fly homolog (Supplementary Table 4, Fig. 6). This observation is important because it suggests that screening for overexpression phenotypes within the collection of UAS-human genes followed by a secondary screen that overexpresses the fly ortholog could quickly identify genes that are uniquely important for specific developmental phenomenon.

While our screen was a success, we would note a few limitations that stem from use of the *GMR-Gal4* driver. It is important to note that, although the *GMR-Gal4* driver is widely employed for gene expression in the *Drosophila* eye, this system is not without its limitations. Although *GMR-Gal4* heterozygotes have not been shown causing visible external defects, florescent staining for apoptosis has been seen in the imaginal disc of third-instar larvae (Kramer and Staveley 2003). Another constraint with utilizing the *GMR-Gal4* system is that expression takes place in cells that have stopped dividing and are differentiating (Freeman 1996). The timing of this expression could exclude genes that are involved in earlier cellular developmental stages.

Based on the literature and results from this experiment, fruit flies are good models to study genes associated with the nervous system and cancer, and our screen results potentially produce new findings for the fly community. The *Drosophila* eye consists primarily of nervous tissue and results from PRECOG showed that many genes in our set were associated with nervous system cancers (Fig. 5). There are several neurodegenerative diseases that are being studied using *Drosophila* such as Parkinson's, Alzheimer's, and Huntington's (Pandey and Nichols 2011). Our screen produced hits that have previously been implicated in these diseases. The gene *β Appl*, which is a fly homolog of the human gene *APLP2*, has been shown to have a protective role in neurodegeneration fly models (Wentzell *et al*. 2012). Another gene includes *APOD*, which in humans has been shown to be elevated in individuals with Alzheimer's and Parkinson's. Function of this gene is neuroprotective with one of its roles being involved as an antioxidant. *APOD* expression in *Drosophila* using a *daughterless*(*da*)-*Gal4* driver increased the lifespan indicating a conserved function across species (Muffat *et al*. 2008). Our new findings from the screen constitute a valuable resource for fly researchers, featuring a greater number of validated conserved genes. Further investigations of these genes may identify additional genes related to neurodiseases and facilitate the development of a *Drosophila* model for studying human diseases.

*Drosophila* has emerged as a powerful model for studying cancer biology (Pandey and Nichols 2011; Katanaev *et al*. 2020). In a genetic screen, where *GMR-GAL4* was used to activate *UAS*-cDNA constructs synthesized from mRNA extracted from human breast cancer tissue, a hit from their screen resulted in a glossy eye phenotype (Katanaev *et al*. 2020). The glossy phenotype appeared similar to the *Glazed* mutant, which has shown to be associated with misexpression of *wingless* (*wg*) (Brunner *et al*. 1999). The Wg pathway is evolutionary conserved in mammals through its homolog Wnt, which is highly studied due to aberrant signaling in this pathway being involved in cancers (Rijsewijk *et al*. 1987; Parsons *et al*. 2021). Katanaev *et al*. discovered that the gene from the breast cancer screen, which led to the glossy eye phenotype, was involved in Wg diffusion (Katanaev *et al*. 2020). As demonstrated in previous studies, the type of mutant eye phenotype observed can indicate a gene's involvement in a specific signaling pathway. Our screen results add more genes to understanding complex signaling pathways. Additionally, our PRECOG analysis offers a comprehensive list detailing the associations between our target genes and various cancer types. The genes are further categorized based on their correlation with either good or poor survival outcomes in cancer patients. In summary, our screen data have identified conserved and functional human genes, which are likely to attract significant interest from the research community. These genes warrant further experimentation to explore their potential roles in oncogenic and tumor suppressor pathways.

## Supplementary Material

jkae188_Supplementary_Data

## Data Availability

All *Drosophila* Stocks are available from the Bloomington Stock Center. The screen results and data from this study are provided in the Supplementary files. Supplemental material available at G3 online.
